# Field testing of user-friendly perennial malaria chemoprevention packaging in Benin, Côte d’Ivoire and Mozambique

**DOI:** 10.1186/s12936-024-04977-0

**Published:** 2024-05-21

**Authors:** Sylvain Landry Birane Faye, Maud Majeres Lugand, André Touré Offianan, Aurélie Dossou-Yovo, Dieudonné Kouakou M’Bra Kouadio, Felix Pinto

**Affiliations:** 1 Laboratoire de Sociologie, Anthropologie, Psychologie (LASAP), Department of Sociology, Cheikh Anta DIOP University (UCAD), Dakar, Senegal; 2 Medicines for Malaria Venture, 20 Route de Pré-Bois, PO Box 1826, 1215 Geneva 15, Switzerland; 3 Department of Parasitology & Mycology, Institut Pasteur of Côte d’Ivoire, Abidjan, Côte d’Ivoire; 4 Directorate of Health Training and Research, Ministry of Health, Cotonou, Benin; 5 Département d’Anthropologie et de Sociologie/Centre de Recherche Pour le Développement, Université Alassane Ouattara, Bouaké, Côte d’Ivoire; 6 Ministry of Health, Maputo, Mozambique

**Keywords:** Perennial malaria chemoprevention, Malaria, Sulfadoxine-pyrimethamine, Packaging

## Abstract

**Background:**

Perennial malaria chemoprevention (PMC) aims to protect children at risk from severe malaria by the administration of anti-malarial drugs to children of defined ages throughout the year. Sulfadoxine-pyrimethamine (SP) has been widely used for chemoprevention in Africa and a child-friendly dispersible tablet formulation has recently become available.

**Methods:**

This qualitative non-interventional observational study was conducted in Benin, Côte d’Ivoire, and Mozambique between February and June 2022. Prototype blister packs, dispensing boxes and job aids designed to support dispersible SP deployment for PMC were evaluated using focus group discussions (FGD) and semi-structured in-depth individual interviews (IDI) with health authorities, health personnel, community health workers (CHWs) and caregivers. The aim was to evaluate knowledge and perceptions of malaria and chemoprevention, test understanding of the tools and identify gaps in understanding, satisfaction, user-friendliness and acceptability, and assess the potential role of CHWs in PMC implementation. Interviews were transcribed and imported to ATLAS.ti for encoding and categorization. Thematic content analysis used deductive and inductive coding with cross-referencing of findings between countries and participants to enrich data interpretation. Continuous comparison across the IDI and FGD permitted iterative, collaborative development of materials.

**Results:**

Overall, 106 participants completed IDIs and 70 contributed to FGDs. Malaria was widely recognised as the most common disease affecting children, and PMC was viewed as a positive intervention to support child health. The role of CHWs was perceived differently by the target groups, with caregivers appreciating their trusted status in the community, whereas health authorities preferred clinic-based deployment of PMC by health professionals. Empirical testing of the prototype blister packs, dispensing boxes and job aids highlighted the context-specific expectations of respondents, such as familiar situations and equipment, and identified areas of confusion or low acceptance. A key finding was the need for a clear product identity reflecting malaria.

**Conclusion:**

Simple modifications profoundly affected the perception of PMC and influenced acceptability. Iterative quantitative investigation resulted in PMC-specific materials suited to the local context and socio-cultural norms of the target population with the aim of increasing access to chemoprevention in children most at risk of severe malaria.

**Supplementary Information:**

The online version contains supplementary material available at 10.1186/s12936-024-04977-0.

## Background

The expansion of access to malaria prevention has had a major impact on reducing the burden of disease globally over the last two decades [1]. However, progress has stalled and children in Africa under the age of 2 years remain at the highest risk of severe malaria and death [2]. Targeted interventions to protect this vulnerable population are needed [1].

Malaria chemoprevention strategies focus on the administration of anti-malarial drugs to vulnerable populations in endemic regions to treat existing infections and prevent new ones [3]. The aims are to protect those most at risk of malaria and reduce disease prevalence. Currently recommended strategies include intermittent preventive treatment in pregnancy (IPTp), seasonal malaria chemoprevention (SMC) to protect children in areas with seasonal transmission during the period of highest transmission risk, perennial malaria chemoprevention (PMC), formerly termed intermittent preventive treatment of malaria in infants (IPTi), and intermittent preventive treatment in school children (IPTsc) [4].

PMC involves the regular administration of anti-malarial drugs to young children in regions of moderate-to-high year-round malaria transmission, regardless of infection status [4]. Sulfadoxine-pyrimethamine (SP) has been widely used for chemoprevention in Africa, including for PMC, and studies have demonstrated its efficacy safety and tolerability [5–11]. Furthermore, pharmacovigilance studies have confirmed the safety of simultaneous administration of SP and vaccines [12, 13], making the Expanded Programme on Immunisation (EPI) a key channel for PMC delivery. PMC has been shown to reduce the incidence of clinical malaria by around 30%, as well as the incidence of anaemia, parasitaemia, and all cause hospital admissions in children aged 12 to 24 months [6–11]. However, SP effectiveness has declined over time, partly due to drug resistance [7], although the presence of SP resistance molecular markers does not necessarily diminish its benefits in chemoprevention [14–16]. Other contributing factors to declining effectiveness include inefficient drug administration, inadequate implementation, or limitations that emerge during scale-up [17, 18].

In Sierra Leone, around half of eligible children received all three PMC doses and coverage was lower than that of concurrent vaccinations [19], highlighting the need to address barriers to uptake beyond non-adherence to the EPI [19].

While PMC was recommended by the WHO in 2010, it has only been adopted in Sierra Leone, where SP is administered at 10 weeks, 14 weeks and 9 months of age alongside the EPI [19–22]. Barriers to PMC adoption have included concerns around dosage and administration to young infants, limited understanding of the clinical impact of molecular markers of SP resistance, and the complexity of the initial policy recommendations [7]. In 2022, the WHO updated the guidance on PMC providing flexibility for National Malaria Programmes in endemic countries to make context-specific decisions regarding transmission intensity thresholds, drug dosages, drug choices and age groups [4].

Typically, SP for chemoprevention is distributed in loose tablet form, which is not particularly child-friendly. Health workers must often crush the tablets and mix them with sugar to make them more palatable, introducing potential dosing errors. Paediatric SP formulations of flavoured dispersible tablets have the potential to enhance acceptability and adherence, while reducing the risk of dosing errors. The recent deployment of dispersible SP for SMC in Burkina Faso showed that peripheral and community health workers greatly appreciated the simplified procedures resulting from the change in drug formulation [17].

The UNITAID-funded project ‘Intermittent Preventive Treatment in infants—Plus’ aims to co-design, pilot, and evaluate country-adapted models of PMC delivered to children up to 24 months of age. This initiative, led by Population Services International in collaboration with the London School of Hygiene and Tropical Medicine, involves government and research partners in four focus countries: Cameroon, Benin, Côte d’Ivoire and Mozambique. The aim is to gather evidence on the impact, effectiveness and cost-effectiveness of the co-designed PMC models to support their adoption and scale-up in sub-Saharan Africa. The Plus Project is designed to work with and within the existing health system structures, leveraging existing points of contact between the health system and children under two years of age to offer SP for PMC. The overarching objective is to inform policy and implementation guidance at the national and global level to support the cost-effective expansion of PMC in sub-Saharan Africa.

To facilitate these pilot programmes, Medicines for Malaria Venture conducted a social research project to develop and validate practical drug packaging solutions and job aids for dispersible SP (12.5/250 mg or 25/500 mg dispersible tablets; S Kant Healthcare, Vapi, India; WHO prequalified April 2021). Drug packaging and prescribing aids represent accessible channels for providing key information about the drug, reassurance regarding its quality, and can support effective prescribing, acceptance and adherence [23–25]. However, such materials must be relevant and appropriate to the end users.

The aim of this study was to support the effective deployment of the child-friendly SP formulation by collaborating with health authorities, health workers, and caregivers to ensure that the proposed drug packaging materials and job aid were adaptable, acceptable, and understandable in diverse settings. The objective of this study was to develop product packaging and a job aid that would promote healthcare providers' and caregivers' understanding of administration procedures and acceptance of the treatment regimen.

Furthermore, the study provided insight on perceptions regarding the role of community health workers (CHWs) in deploying the new intervention. These objectives align with global efforts to combat malaria and improve health outcomes in malaria-endemic regions.

## Methods

### Study sites

The study was conducted in Benin, Côte d’Ivoire and Mozambique between February and June 2022 across nine sites, three from each country. These were selected either randomly or purposely, depending on the context, to include health districts with a significant malaria prevalence in children under 5 years old and to reflect the diversity of settings in each district.

In Benin, the study was performed in the Tanguieta-Materi-Cobly health zone in north-western Benin. In each commune two arrondissements were randomly selected: Tanguiéta (Tanguiéta, Taïacou) Matéri (Matéri centre, Tchanhoun-Cossi) Cobly (Cobly centre, Kountori). The climate is Sahelian and malaria prevalence during the dry season (November to April) is 50%, increasing to 78% during the rainy season (May to October) [26]. CHWs interface between health facilities and populations, delivering health promotion in the community. SMC and IPTp have been implemented. PMC was piloted in 2007 via the EPI by UNICEF as a research project to assess feasibility, but the intervention was not adopted [27].

In Côte d’Ivoire, the study was conducted in the health district of Agboville, located in the Agency-Tiassa-Me region (South). Three sites were purposely selected to represent the different health services available: Azaguié and Grand-Morié have Urban Health Centres, whereas Agboville has a Regional Hospital Centre, a Maternal and Infant Protection Centre, and additional peripheral health centres. Malaria is endemic throughout the year and malaria prevalence in children under 5 years of age of was 70% in 2020 [28]. Public health is supported by a recently formalised community sector. Côte d’Ivoire is piloting PMC implementation via EPI services in 10 target health districts.

In Mozambique, the study was conducted in all three districts of Mocuba in Zambézia province (Centre), i.e., Mocuba Sede, Namanjavira, and Mugabe. The area has high malaria transmission, with an RDT-based prevalence of 68% in children under five in 2015 and up to 47.8% in children below 15 years [29, 30]. The primary level health system consists of health posts, health centres and rural hospitals. Mozambique has implemented IPTp and began SMC implementation in 2020 [31].

### Study design

This qualitative, non-interventional observational study used focus group discussions (FGDs) and semi-structured in-depth individual interviews (IDIs) with key implementing partners, i.e. health authorities and Plus Project in-country teams, service providers, and caregivers to evaluate prototype packaging for SP blisters and a dispensing box as well as a job aid. Perceptions and knowledge of malaria aetiology and prevention and the acceptability of PMC deployment through CHWs were also explored.

### Study materials

SP tablets were previously supplied loose in jars, whereas the child-friendly dispersible formulation will be supplied in blister packets. Blister packaging provides some reassurance of quality and protection for the drug from contamination, degradation and damage. A blister pack has individually sealed tablets which must pushed through a foil cover to take the medication, it is tamper-evident and can help with adherence as it can easily be seen whether the previous dose was taken [23, 25]. A dispensing box can reassure caregivers that the blister packs are genuine medicine and can also serve as an educational aid both as a reminder of the procedures for administration for the health worker or CHW and to help inform caregivers about the medication [24]. The job aid aimed to provide clear instructions to health workers and potentially CHWs regarding PMC, including its meaning, components, eligibility assessment, SP administration linked to the vaccination calendar, adverse effects, consultation timing and location, medication timing and storage.

Designs for these materials were initially developed by a graphic designer based in Geneva. The FGDs and IDIs tested the understanding of the tools by the partner groups to identify gaps in understanding, satisfaction, user-friendliness and acceptability. Results of the tests were analysed with local teams and participants daily and shared with the graphic designer remotely. Continuous comparison across IDIs and FGDs permitted evaluation and iterative development of materials. Updated versions were generated for retesting and validation, with researchers interacting directly with participants and collating their views on how the tools could be improved. At the end of the testing, preliminary results were shared with the National Malaria Programme and representatives from partner non-governmental organizations.

### Recruitment

The study target groups were caregivers with infants < 24 months of age living in the selected locality (men, women, older women), CHWs living in the selected localities and engaged in health promotion and community-based activities for malaria, health professionals (nurses, midwives, health assistants) in the selected localities engaged in malaria or maternal healthcare, and health authorities at the district level responsible for the malaria programme (health district medical officers). All study participants were over 18 years old. Caregivers without infants and/or not living in the selected localities were excluded, as were health workers and CHWs not engaged in malaria interventions or community-based health promotion activities for malaria.

### Data collection

Overall, 106 target persons took part in IDIs and 70 in the FGDs (Table 1). FGDs included five to eight participants for each target group, i.e. CHWs, maternal and child midwives and preventive medicine officers (Table 1). IDIs were conducted using three specific interview guides for caregivers, CHWs/health professionals, or health authorities (Additional file 1).

**Table 1:** Distribution of participants surveyed by in-depth individual interview and focus group discussions

In-depth interviews	Côte d’Ivoire	Benin	Mozambique	Total
Caregivers	9	13	12	34
Community health workers	11	11	13	35
Health personnel^a^	9	7	8	24
Health authorities	5	6	2	13
Total	34	37	35	106

Focus group discussions	Côte d’Ivoire (3)	Benin (6)	Mozambique (3)	Total (12)
Community health workers	16	16	7	39
Maternal and child midwives	0	0	8	8
Preventive medicine officers	0	16	7	23
Total	16	32	22	70

^a^Nurses, midwives, and health assistants

Initially, general questions explored knowledge and perceptions of the importance of malaria in the community, who was most at risk, and preventive measures against malaria, including chemoprevention and specifically PMC. The purpose of PMC was then explained, and participants questioned as to their perceptions of the intervention and the role of CHWs in administering PMC. Participants were then provided with the relevant prototype for the blister pack (tablet-free), dispensing box and job aid. First, the prototypes were shown to the participants without introduction or preamble to not bias the response. Their initial reactions, impressions and understanding were collected. Second, after explaining the ideas conveyed by the tools, participants were asked whether the images and designs accurately represented the ideas, and their impressions on what improvements and amendments were required. Then participants were asked to explore the tools again based on specific scenarios, including comparing different versions of the tools, and to explain their preferences. Research notes were taken from direct observations of the participants’ reactions while capturing their speech, attitudes and verbal and non-verbal exchanges during their initial examination of the materials. Finally, participants were asked specific test questions regarding their impressions on the importance and usefulness of the tools.

### Data analysis

All direct observations were recorded and incorporated into the analysis. Interviews and FGDs were transcribed using a labelling system to facilitate their management and treatment. For quality control, 20% of transcripts were compared with the audio recordings. Transcribed data were imported to ATLAS.ti (ATLAS.ti Scientific Software Development GmbH, Berlin, Germany version 20) for encoding and categorisation. Hybrid inductive and deductive thematic content analysis was used [32, 33]. Themes were initially generated from the data using deductive coding based on the general knowledge/perceptions regarding malaria and chemoprevention, and understanding and appreciation of the tools, complemented by inductive coding based on new themes from the observations and transcripts [33]. Results were triangulated according to the individual tools and target participant groups within each country. By cross-referencing the views concerning a theme between countries, data interpretation was enriched, and country similarities and differences highlighted.

### Ethical approval

Ethical approval was obtained from the national ethics committees of each country, i.e., Benin, National Health Research Ethics Committee (NHREC), Cotonou, Akpapka (authorisation 48, November 30, 2021); Côte d’Ivoire, National Health and Life Sciences Ethics Committee (NHLEC), Abidjan, Côte d’Ivoire (IRB000111917); Mozambique, Comité Institucional de Bioética do Instituto Nacional de Saúde (CIBS-INS), Maputo (authorisation CIBS FM&HCM/095/2021). The protocol was also approved by the WHO Research Ethics Review Committee (ERC.0003673). In addition, preliminary meetings were held with the district health authorities and the chiefs of the concerned localities to present the content of the survey, obtain their commitment and assistance, and ensure data collection in the field. All participants provided written consent for use of their de-identified contributions.

## Results

**Fig. 1:**
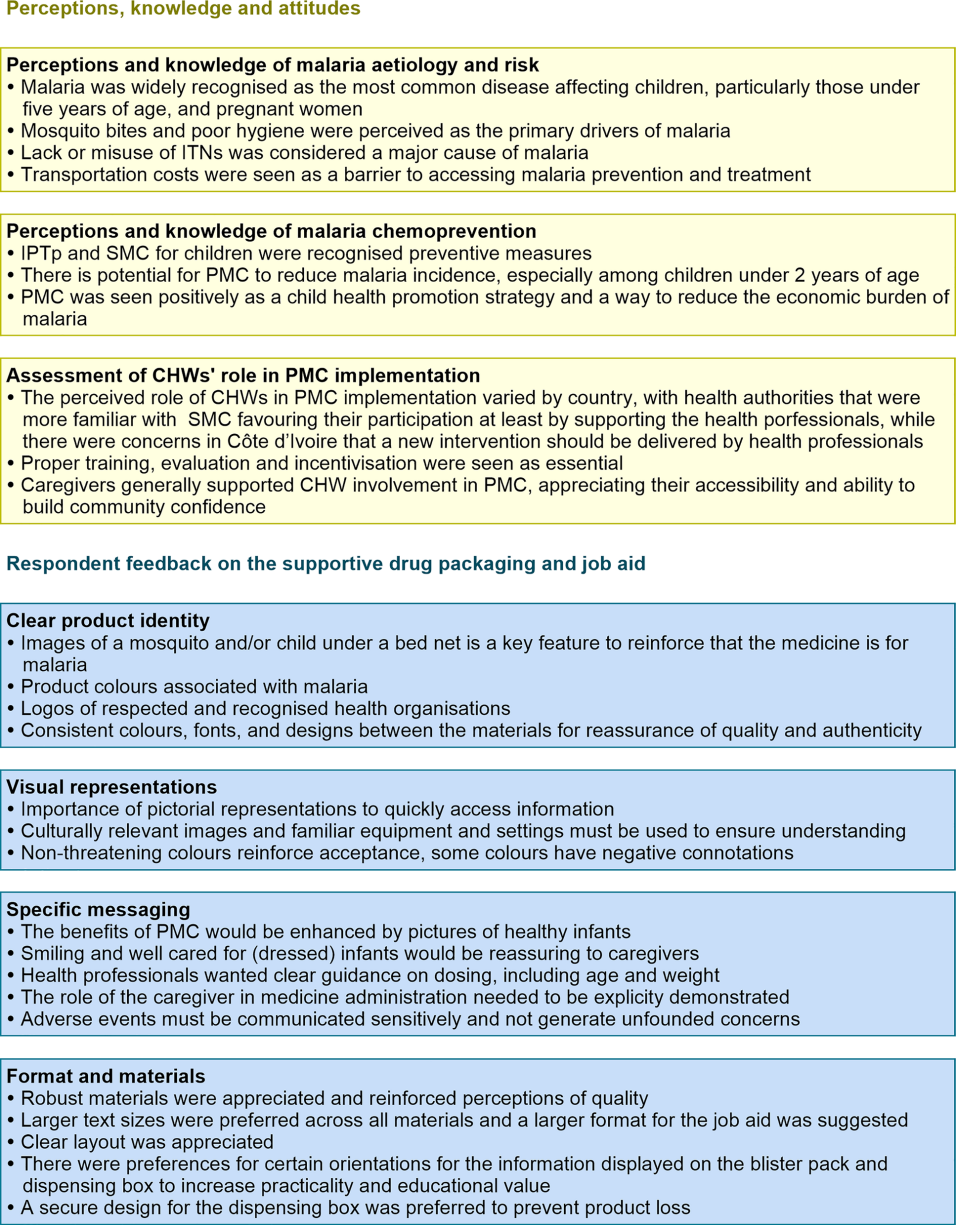
Summary of findings

An overview of the key findings of the study are shown in Fig. 1.

### Perceptions and knowledge of malaria aetiology and risk

Participants across the three countries considered malaria the most common disease in children, posing risks such as anorexia, diarrhoea, high fever, seizures, anaemia, malnutrition, failure to thrive, abortion, vomiting and death. Children under five, especially those whose parents are without formal education or who have poor understanding of malaria, and pregnant women were thought to face the highest risk. Caregivers associated transportation costs as a key factor in limiting access to malaria prevention and treatment.

*The most recurrent diseases among children in the health district of Agboville are malaria first, followed by diarrhoea and ARI [acute respiratory infection]. The age group of children affected is 0 to 5 years old.* IDI_epidemiological surveillance supervisor_Côte d’Ivoire*.*

Overall, there was a consensus across all respondents that insecticide-treated bed nets (ITNs) were the most effective way to safeguard children from malaria along with environmental hygiene awareness and good housekeeping. CHWs and caregivers also recommended using repellents (insecticides, window screens, house sprays, coils or mosquito nets), herbal teas, prayers for divine protection, indoor insecticide spraying, burning eucalyptus leaves and limiting children's outdoor exposure at night. In Benin, exposure to the elements, poor food hygiene and alcohol consumption were mentioned by some CHWs and caregivers.

*Here, mosquito nets are distributed, so people sleep downstairs to protect themselves from mosquito bites. However, when parents do not have the means, they sometimes use traditional medicine.* IDI_community health officer_Côte d’Ivoire.

*The causes of malaria, first of all, we will talk about the mosquito bite. When someone does not sleep under an impregnated mosquito net, the person can get malaria. After that, there is also the sun and the rain. Children are fragile to these causes...* FGD_CHW_Benin.

### Perceptions and knowledge of malaria chemoprevention

In all three countries and across the respondents, IPTp was known for protecting pregnant women from malaria and in Benin, SMC was known to protect older children from malaria. While PMC was unfamiliar to respondents across all countries, following explanation of its objectives, it was perceived as a valuable addition to malaria prevention efforts. Respondents recognised the potential for PMC to reduce malaria incidence, which is the main reason for local health consultations among children. They viewed PMC positively as a child health promotion strategy, noting the potential to address neonatal and infant malaria cases resulting from gaps in IPTp and ITN use. Health authorities and health professionals considered coupling PMC with routine immunization an opportunity to reduce vaccine hesitancy and strengthen the EPI. In Benin, where SMC is already implemented, respondents believed that adopting PMC could reduce the overall burden of malaria. In Côte d’Ivoire, potential challenges associated with directly observed treatment (DOT) were raised by health authorities, as they had experienced similar limitations with IPTp. In contrast, across all countries CHWs considered PMC a promising addition to existing malaria prevention methods and caregivers believed PMC could benefit families by alleviating the economic impact of the disease.

*SMC alone is not enough to eliminate malaria. If the children take the PMC at the hospital, it is good, but to save those who do not come, we need the help of the CHWs to go door to door.* IDI_caregiver_Benin.

*Now, in the real implementation, we will know its shortcomings to improve them. It is a bit like IPT for pregnant women. When we started the DOT strategy, there were some challenges to overcome, and a little information was passed on to the National Malaria Program. This is also an important aspect reinforcing the classic measures already in place. If we administer SP in infants with the distribution of LLINs [long-lasting insecticidal bed nets], I think one in the other, the strategy is good in terms of strengthening*. IDI_head of civil society organisation_Côte d’Ivoire.

*Way you explained it is terrific. It is going to help the children even. It will help the parents [...]. Right now, everything has become expensive. So, if there is medicine to help the children, it is good. At least we will not spend too much on children's illnesses*. IDI_ caregiver_Côte d’Ivoire.

### Assessment of CHWs’ role in PMC implementation

Health authorities positions on CHW involvement in PMC varied by country. In Côte d’Ivoire, distribution by qualified health professionals was preferred, owing to the strategy's newness, with CHWs only working under health worker supervision. Concerns included CHWs' volunteer status with the extra work potentially hindering routine activities. In contrast, in Benin CHW involvement in PMC was well-received by health authorities and it was thought that CHWs could also assist overwhelmed vaccinators, improving acceptability among parents. For Mozambique, CHWs were viewed as valuable for PMC implementation, as their door-to-door approach could identify reluctant individuals and those lost to follow-up and their community acceptance and familiarity with drug distribution were highlighted.

*Well, the CHWs, I do not see the relevance of their place in the activity. Unless, at the vaccination station, someone is delegated to give the medication.* IDI_health authority_Côte d’Ivoire.

*Entrusting the PMC to the CHW is even the best solution because they represent the population in the villages. They are the ones who drive almost everything. So, if they could come here (health centre), it would allow the population to be more accepting and reduce the workload of the immunisation health agents.* IDI_health agent_Benin.

*It is feasible because in the community, we have CHWs, and they are distributed in almost all health areas, although some villages are not yet covered. It would be good with instructions they will manage in the community that they would monitor, any reaction report to the district health unit or the provincial health directorate or ministry of health.* IDI_health authority_Mozambique.

Although positive about the role of CHWs in health promotion, health workers were concerned that CHWs did not have the skills to take charge of PMC as a routine activity. There were doubts whether CHWs could follow instructions and maintain hygiene requirements and proper training and evaluation were thought essential. As expected, CHWs desired full involvement, emphasizing their role in distribution and community mobilization, though financial incentives were seen as important to maintain motivation.

*We are the first in the villages. Even with all the SMC activities, we are the ones who do them. We are used to this kind of activity. Some mothers do not like to go to the health centre. In these conditions, if the infant's SP is with the CHW, her baby will benefit easily.* FGD_CHW_Benin.

Caregivers favoured CHWs involvement in PMC, appreciating their authority and ability to build community confidence. In Benin, caregivers welcomed activities preventing infant illness and the reduced need for health centre visits which are time consuming and incur costly travel expenses. However, concerns included potential adherence issues if CHWs left the product at the house without DOT and worries about CHW training and skills. In Mozambique, some parents cited hygiene concerns and side effects as reasons for preferring health facility administration.

*If they are the ones who will always distribute the SP tablets, it is better for us because they come to our houses to give them to the children, the expenses that you used to make to go to the hospital, you will not make them anymore. So, it is a good job.* FGD_grandmother_Benin.

*I prefer that the product be given to us at the health centre because we take the products without hesitation in front of the health worker. With the CHWs, we can keep it and when they leave, not take it.* IDI_parent_Benin.

### Empirical test of SP blister prototypes

**Fig. 2:**
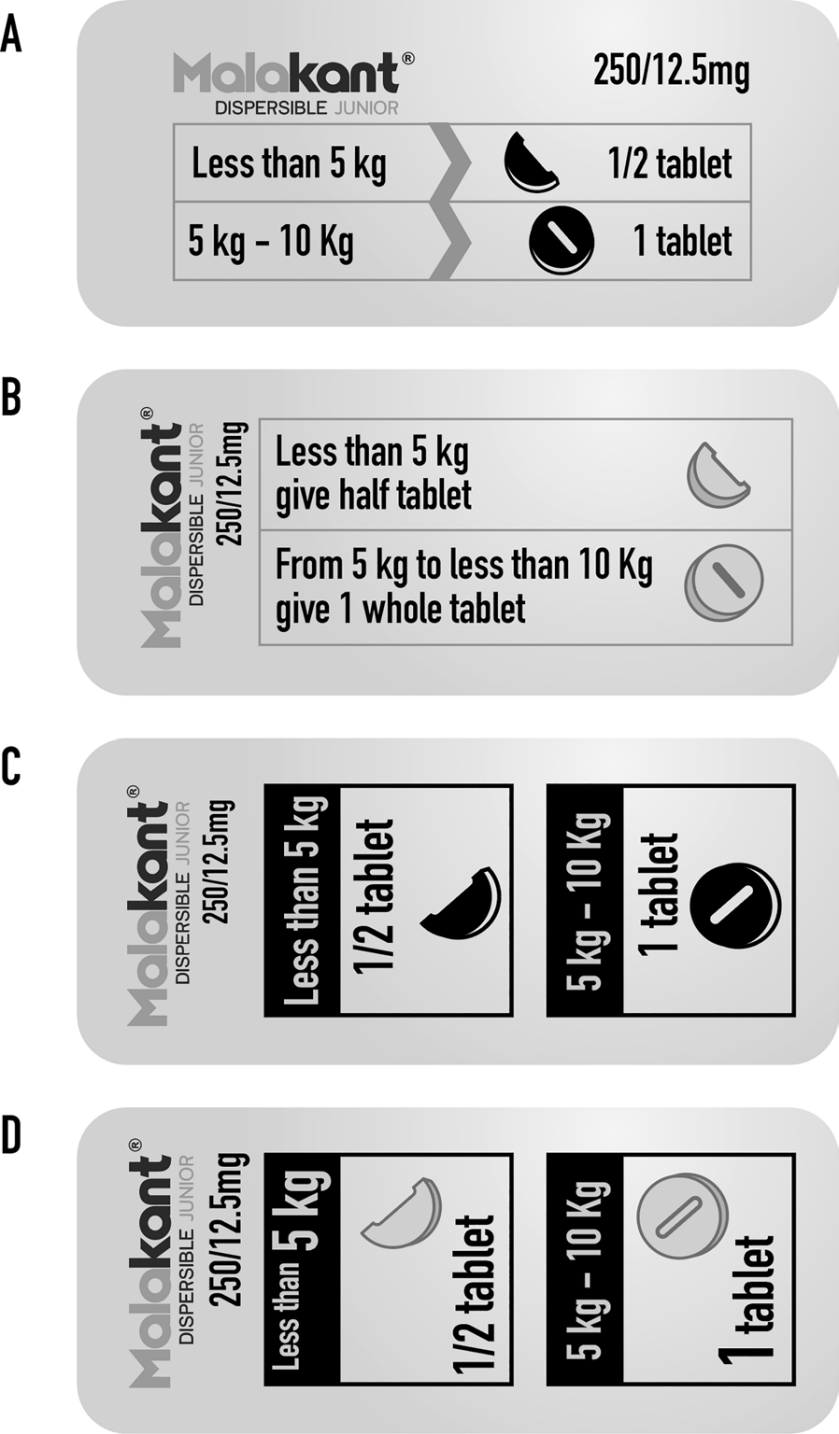
Versions of the SP blister pack tested. Three versions (**A**–**C**) of SP blister pack tested in Côte d’Ivoire and Benin and an improved version tested in Mozambique (**D**)

Three initial versions of the blister packaging prototype (A, B and C) were tested across all four respondent categories in Côte d’Ivoire and Benin for suitability, practicality, and presentation (Fig. 2). Among the three formats, the vertical blister (C) received the most favourable responses due to the layout of information. The feedback from these assessments was used to create an improved SP blister tested in Mozambique (D), which was deemed acceptable, well-designed and easy to understand (Fig. 2). All respondents gave positive ratings to the blisters, noting their overall presentation, legible typography, and inclusion of dosage recommendations directly on the blister, though further enhancements were suggested.

In all countries, the main proposed improvement from all respondent categories was the colour of the SP image of the blister. Black was not acceptable, with both caregivers and health authorities suggesting a coloured tablet, such as yellow (associated with malaria) and white (cheerful), matching the usual colour of SP from health services, or pink (child-friendly). Note that the manufacturing process for the blister packs is limited to black printing on a white background, so adoption of other colours was not feasible. However, a white tablet, was considered acceptable (Fig. 2D).

*… the colour of the medicine is not attractive if we could adapt it to the colour of the SP package that was presented to us to have a mix of colours that resembles that of the package. Because for the campaigns here, it is the yellow colour that the SP drugs have. The rest is fine.* IDI _ health officer_Benin.

Both caregivers and health authorities suggested a picture of an infant or a mosquito to reinforce that this was an anti-malarial drug for children. Caregivers were concerned that the drug should have a sweet taste. Caregivers found the information easy to understand but suggested enlarged images and modified text formats. Health authorities and health professionals understood the dosage and administration information well but also recommended presenting dosing options for both age and weight. CHWs found the format easy to understand but insisted on including the dosage:weight ratio and complete dosage information on the blister pack, especially for children over 10 kg. Notably, in Benin, where health workers have experience in SMC and IPTp, technical enhancements were proposed, including making tablets bi-coloured to indicate their composition, ensuring tablets are easily divisible, emphasizing ‘dispersible’, and presenting dosing options based on age and weight. They also suggested using the same colours on the blister pack as the external packaging so that users know that they are receiving the medicine, which is in the packaging.

*Add a picture of a mosquito on the blister to reinforce the identity of the drug so that even if they do not have the package, they know what the drug treats.* IDI_healthcare provider_Benin.

*I see 0 to 5 kilo, ½ tablet, 5 to 10 kilo, one tablet. Here I think the writing is fine. It is good because the writing is accurate. They put half a tablet and one tablet. Even if you do not know how to read, you can already see what it looks like.* IDI_CHW_Côte d’Ivoire*.*

### Empirical testing of the dispensing boxes prototypes

**Fig. 3:**
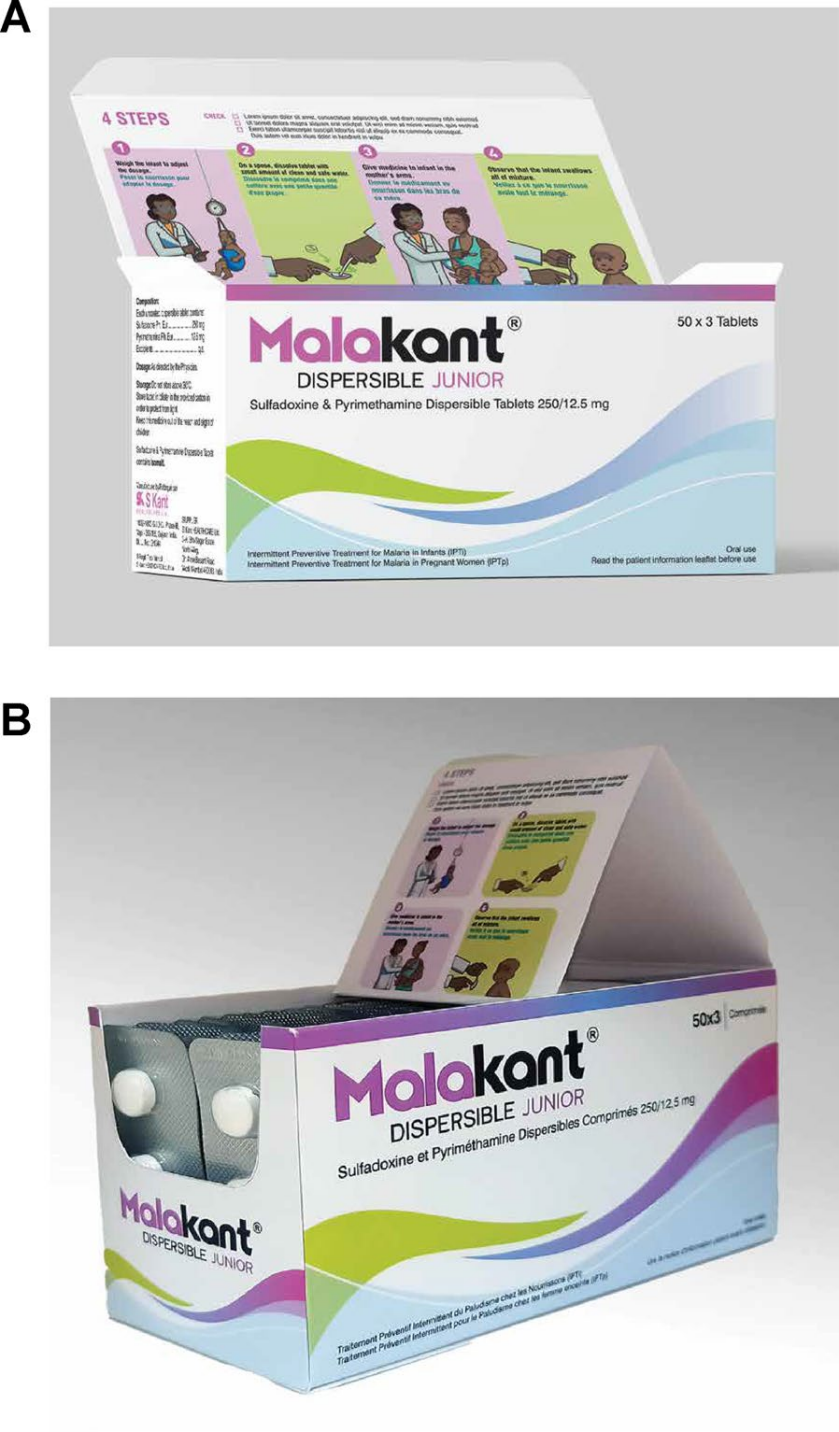
Two versions of dispensing boxes tested in the three countries

Two versions of the SP dispensing box were evaluated across all countries, each presenting information differently (Fig. 3). Respondents gave positive feedback on the dispensing box, including the use of bright, non-aggressive colours, image presentation, and paper quality. Health authorities and medical staff appreciated the information and recommendations on the dispensing box as a helpful reminder for drug administration.

In terms of the format, most respondents of all categories preferred version 1 as this was considered secure, allowing blister conservation and minimizing the risk of product loss (Fig. 3A). In contrast, version 2, which opened sideways, had lower-quality paper, making it harder to open and potentially causing blister loss due to a lack of reinforcement (Fig. 3B). The horizontal presentation of information on version 1 was also generally preferred, aiding understanding and implementation of recommendations, including for those without formal education. However, some respondents preferred the information on the top cover as in version 2. Respondents suggested presenting information more pictorially and enhancing the product identity with images like a mosquito, child, or a child under an ITN as well as increasing the text size. Additionally, they preferred having illustrative images on the outer sides of the box for quick access to information, which would enhance its role as an educational tool (Fig. 3).

*I chose the box with the top opening because it is well protected. When you close it, it stays closed, safe, and well protected. The medicines cannot spill. Whereas the other one, the closure is not solid. Medication can easily spill. So, I choose the first box.* IDI_primary healthcare centre_Côte d’Ivoire.

*Overall, everything is clear except for the missing pictures that need to be added. The dosage is suitable, the preparation too, on how to give, and everything is clear for me. Nonetheless, you could add a picture of a mosquito or a baby lying under a net at the top of the brochure to show that it is for malaria.* IDI_CHW_Benin.

**Fig. 4:**
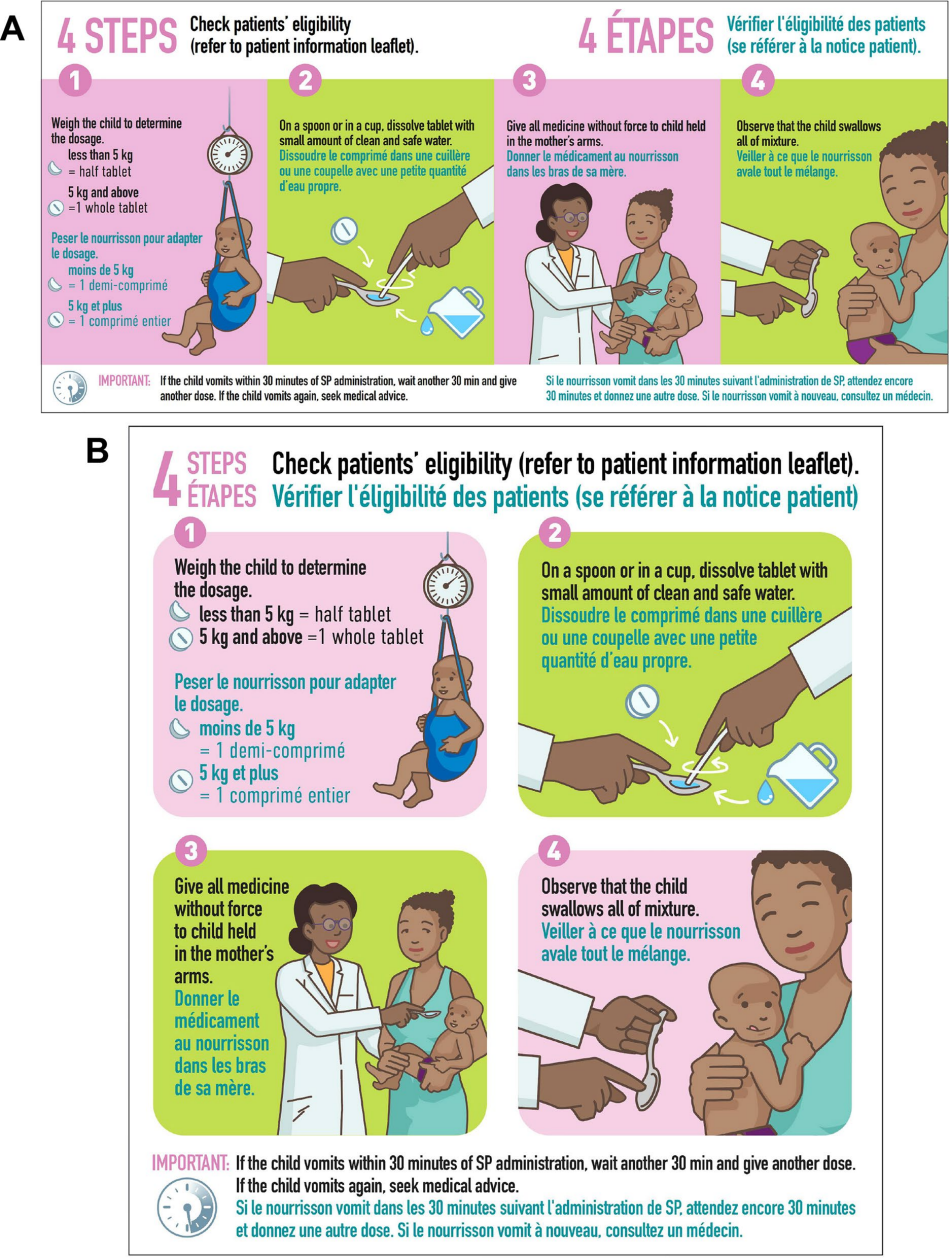
Dispensing box information for prototypes 1 and 2

The educational content on the dispensing box was assessed by examining how respondents understood dosage and administration information, including steps for verifying a child's eligibility and administering SP, presented differently in two prototypes (Fig. 4). Health workers and CHWs in all three countries were able to decipher the information but suggested specific enhancements. For step 1, commonly used weighing scales were needed. In step 2, they recommended replacing the spoon image, which is associated with crushing tablets, with an image of a hand holding a glass with a small amount of water or a small measuring cup and another hand dropping the tablet into the glass, to indicate a dispersible medicine. For steps 3 and 4, respondents noted that the nurse as the central figure did not reflect reality, especially in paediatrics. Caregivers believed that the mother should be involved in the administration, holding the child, giving the medication and providing reassurance.

*Also, I would have preferred that the water not be coloured blue, so it does not look like the tablet has turned that colour. Also, I would prefer that the cup be moved up and that the child who has finished taking the product faces the spoon and the health worker rather than having his back to the spoon because it could be thought that it is because the child refused the product that he grabbed his mother's breast. The child must also be smiling to show that you are in good health when you take this product.* IDI_caregiver_Benin.

### Empirical testing of the job aid

**Fig. 5:**
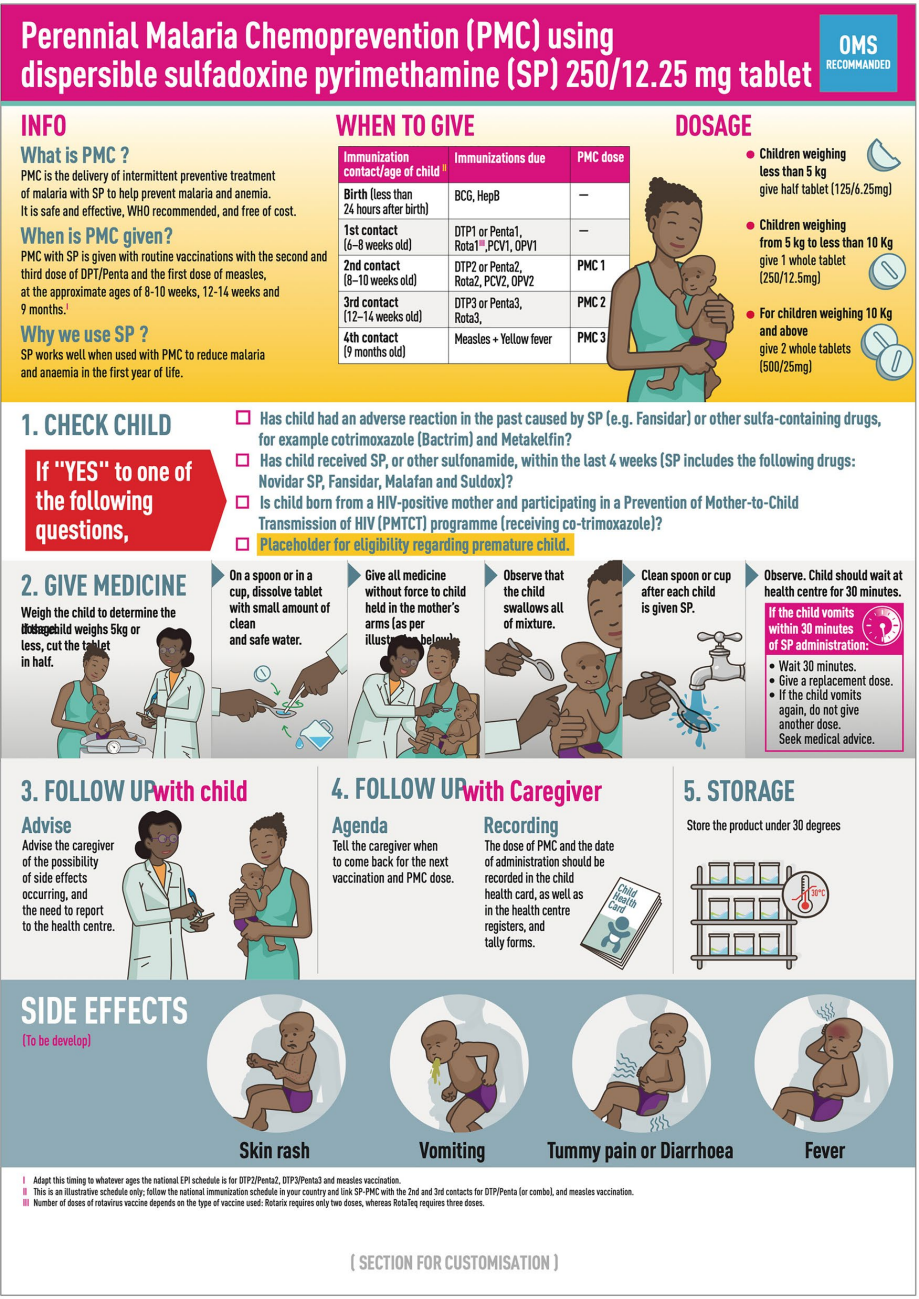
Job aid version tested in Côte d’Ivoire

**Fig. 6:**
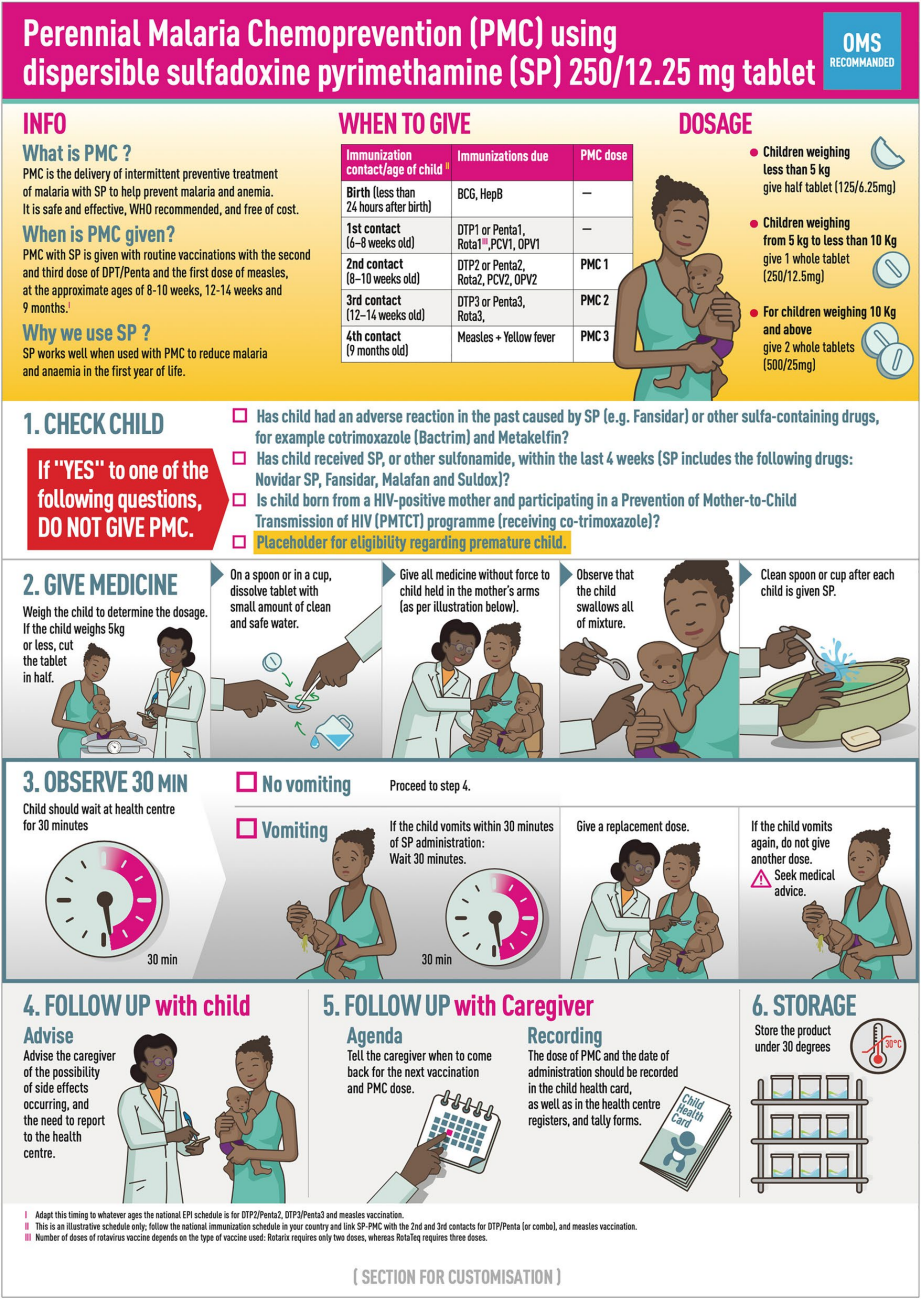
Job aid version tested in Benin and Mozambique

The initial version of the job aid was tested in Côte d’Ivoire (version 1) (Fig. 5), and findings incorporated into version 2 for testing in Benin and Mozambique (Fig. 6). The key differences were that version 1 included a single image relating to the observation time following administration, and an additional block on potential adverse events. Version 2 included a more extensive pictorial representation of the 30-min observation period after SP administration, including what to do if the infant vomited, without the separate section on adverse events. Overall, respondents found the job aid useful and comprehensible, particularly appreciating the graphic elements for aiding communication for those with lower literacy. CHWs found it a helpful reminder for an unfamiliar treatment, while caregivers were drawn to the images.

*It is the information about the drug. The images show how to administer the drug. First, there is information on PMC: its definition, when to administer it, and dosage. Then there are the contraindications, the drug's process, and finally the side effects*. IDI_health authority_Côte d’Ivoire.

In terms of the format and overall presentation, some respondents expressed dissatisfaction with the small text size on the A3-sized paper and suggested a larger format for better readability and understanding. Respondents from all categories recommended adding images of a mosquito and child sleeping under an ITN for product identification. In addition to the WHO logo, health authorities suggested adding the Ministry of Health, Public Hygiene logo to enhance user confidence in the information presented.

The first section of the job aid included general information on PMC, when it should be administered, and dosing and was similar between the two versions. Instead of a mother holding a semi-clothed infant, health workers preferred a picture of a mother with a healthy, fully dressed infant to emphasize improved quality of life. For caregivers, a picture of a woman with an infant in her hand reinforces that SP is for the baby this time. In Benin, where health workers and women were already familiar with IPTp, it was suggested that a picture of a pregnant woman holding three SP tablets and pointing to the picture of a woman holding an infant would illustrate that PMC is specific to infants. Posology was clear and appreciated, with suggestions to use baby images lying down, sitting, and standing to represent tablet dosages. It was also mentioned that the dosage illustration should align with packaging and blister information. Some asked if child weight alone or weight and age should be considered. In Mozambique, the timings for PMC needed to align with the national immunisation schedule.

*I would like to see a drawing of a mother with a 10-week-old child, for example, and put the dosage in front of him. We put his dosage in front of the child, who is already 14 weeks old. If it is a child crawling, put the dosage in front of him, the child who is nine months old, put the dosage in front of him, and so on. Nevertheless, there, too, it can be a mistake.* IDI_health worker_Benin.

There were no specific comments on assessing eligibility, but illustrations for contraindications were suggested. In Benin and Mozambique, HIV-related concerns arose, and respondents suggested not providing PMC in case of doubt and adding ‘Do not administer this medicine to a sick child’. In Mozambique, health authorities were opposed to the suggestion of adding the HIV logo, as it may be off-putting to caregivers.

The section on medication administration received specific comments on equipment and procedures. Similar to the dispensing box, images related to the baby scale, dissolving tablets in a spoon, and the mother's posture needed revision. Respondents suggested using a measuring cup, a sitting mother in a breastfeeding posture, and the health worker wearing a headdress, as is usual. Caregivers suggested dressing the child in shirt and underpants after it had been weighed because parents felt that an infant should not remain shirtless for a long time.

*The brochure details everything on package one that I chose. The messages on how to give SP are well presented and understood but still need the mosquito drawing on the brochure. IPTi will be included with routine immunisations listed on the immunisation schedule. If the child takes the medication and vomits thirty minutes later, it must be given again. However, is this how you keep an infant? The baby in the picture does not show that it is an infant.* IDI_health agent_Benin.

The half-hour observation period after ingesting the product and the procedure was effectively conveyed by the clock image. However, some respondents suggested alternative representations of the time passed. There was concern that the 30-min waiting time could hinder the initiative's success, given that this is in addition to the time caregivers wait to be seen. Reducing the observation time to 15 min as per SMC would increase acceptance. Vomiting images needed adjustment and were seen by health workers as an opportunity to educate on the correct way to hold a vomiting child. Additionally, an image pointing to a doctor or health centre was needed for if the child vomits a second time. All respondents agreed on the need to illustrate adverse effects to fully inform communities about the medication, but some raised concerns about creating unwarranted fears. Also, there was some confusion on version 1 regarding the illustration of itching and health workers wanted abdominal pain and diarrhoea to be distinguished from each other as they may not occur together as shown in the picture. Health workers required a final line on what to do if adverse effects occur.

*You know that our parents do not use watches for waiting time. They use the sun much more to define time. A watch would be good if we could bring out a natural effect. The image of a health worker welcoming the mother and her child should be used to manage side effects.* FGD_caregiver_Benin.

*Well, I saw the pictures, and they wrote things down. So, I see the child has given medicine to the health worker, a scale, and the health worker advises the mother—about the effects of malaria. The child is cold and has diarrhoea, vomiting, and fever.* FGD_CHW_Côte d’Ivoire.

For the section on follow-up, the first image was perceived to be the health worker providing advice to the caregiver on adverse events. Health workers suggested including the health worker's gestures and speech bubbles to clearly illustrate this. For the images related to appointment dates and immunization records, the booklet image was considered inadequate and mistaken for a notebook or mobile phone. It was recommended that the words ‘Mother–Child Health Record’ be used. In Mozambique, the image should match the immunisation booklet. Understanding the image on medication storage was facilitated by a crossed-out thermometer, but it could be improved with a window door and locker representation.

*Where the provider is informing the mother about the occurrence of side effects, is confusing. The information conveyed is not perceptible. It looks like the provider is mentioning the date of the next appointment in the notebook since he is holding a notebook and a pen. What would be interesting is to draw bubbles next to the provider's mouth and write the message inside that bubble like we usually see with comic books. It reflects an interaction.* IDI_head of civil society organisation_Côte d’Ivoire.

## Discussion

In sub-Saharan Africa malaria is the leading cause of mortality and morbidity in children under 2 years of age, and PMC has the potential to decrease the incidence of malaria and reduce hospitalisations in this population [20]. However, experience of SMC implementation suggests poor adherence to chemoprevention can result from failure to appreciate the benefits of the medication, inadequate explanation of medication administration, concerns regarding adverse reactions and not considering caregivers living conditions, such as lack of access to clean water [34].

Benin, Cameroon, Côte d’Ivoire and Mozambique are piloting PMC implementation to inform effective deployment strategies. A part of this process, this study investigated the perceptions, knowledge, and practical aspects of malaria chemoprevention among health authorities, health providers, CHWs and caregivers in Benin, Côte d’Ivoire and Mozambique. Specifically, it conducted empirical testing of prototype PMC-specific blisters and dispensing boxes for dispersible SP and a job aid aimed at supporting medication delivery, administration, acceptance and adherence. Additionally, it assessed the potential role of CHWs in distributing PMC.

Across all three countries, malaria was perceived as a prevalent disease, which threatens children, the wellbeing of families and community development. Children under 5 years were thought to be most at risk, though older children and adults were also believed to be at risk where SMC is deployed. This perception is supported from evidence showing a shift of malaria risk to older children in some SMC areas [35]. The causes of malaria were well understood by carers, CHWs and health workers and preventive measures such as ITNs were widely known. These findings are consistent with studies in African countries with high malaria prevalence [36–40]. However, respondents were aware of a lack of knowledge and understanding within certain sections of the community, such as those without formal education. Perceptions and knowledge of malaria aetiology and risk, and more generally health literacy, have been shown to influence the adoption of chemoprevention by caregivers [34, 40, 41]. For SMC, sensitisation and education efforts directed at caregivers were associated with improved coverage [41]. In Sierra Leone, children who slept under an ITN the previous night were more likely to have completed the full course of PMC [19], suggesting that those caregivers who are already taking measures to protect their children from malaria are more likely to accept additional interventions with the same purpose.

Across all three countries, awareness of PMC was low, but following explanation of the aims of the intervention acceptability was high in all respondent categories. Previous pilot studies in Francophone and Anglophone countries in Africa have reported that good awareness and high acceptance of PMC can be achieved across health professionals, CHWs and caregivers, though educational interventions were required to address some misconceptions [27, 42]. In Sierra Leone, there was no indication that PMC negatively impacted attitudes towards or adherence to the EPI, or the pursuit of treatment or existing malaria prevention measures [43].

By delivering PMC through the EPI, the existing infrastructure can be used, and the number of contacts with the health system minimised for caregivers. Not only does this reduce costs for the health system, but it also reduces the cost to families for transportation and time off work compared with having to make a separate visit to the clinic. A review of contextual factors affecting the uptake of chemoprevention measures found that travel distances and inclement weather limited both SMC and PMC access [41]. In Sierra Leone, completion of the PMC course was independently associated with the child’s caretaker being in paid employment, suggesting that families that are subject to greater financial pressures are less likely to access this intervention [19]. In the current study, the potential financial benefits of PMC in reducing the risk of malaria were understood by caregivers. Also, caregivers who were familiar with SMC were more accepting of CHW door-to-door delivery of PMC, with one of the perceived advantages being the avoidance of transport costs to the clinic.

There was a preference among health authorities for PMC being delivered at health centres via the EPI, though previous experience with SMC was associated with a more favourable disposition to CHW involvement in PMC. Health professionals emphasized the need for sufficient training and supervision of CHWs, whereas CHWs highlighted the need for incentivisation. Caregivers in this study were more accepting of PMC when delivered by CHWs as trusted individuals, and parental trust has been identified as a key driver of uptake of SMC [44]. If PMC is delivered via EPI visits, families who do not participate in the EPI will also not participate in PMC unless additional outreach activities are done. Some health authorities thought that CHWs could play a key role in simultaneously supporting the EPI and PMC in accessing these families. Notably, in Sierra Leone, uptake of PMC was lower than for EPI, but CHWs have not been mobilised to support the programme [19, 22]. In contrast, SMC is delivered in the community by CHWs, and door-to-door delivery was associated with high coverage rates and improved caregiver acceptance [41, 45, 46]. Conversely, sub-optimal uptake was associated with insufficient CHWs, time constraints, missed households or settlements, parental reluctance, child sickness, and child absenteeism [17, 21, 44, 47]. Rather than vertical delivery of PMC through CHWs, there may be potential synergies in the integration of CHW involvement with the EPI, though this has not been tested in a field setting. However, there is some evidence of this integrated approach from Nigeria where CHW referrals of pregnant women for ante-natal care and IPTp resulted in a 60% increase in the probability of women receiving all three doses of IPTp [48].

The potential for drug packaging to be used to support effective prescribing, acceptance and adherence has been a neglected aspect of chemoprevention implementation. Blister packs have been shown to improve drug adherence generally, but the impact of including prescribing information on blister packs is less studied [25]. However, this was previously evaluated in a quantitative study testing perceptions of drug packaging SP for IPTp, with respondents reporting reassurance that the drug was genuine, had not expired, and understanding that the drug was for pregnant women [24]. The current study reported similar enhancements of user perceptions, with ideas around quality, the facility to double-check the dosage, and the targeting of the drug at infants.

Reassurance on the value of PMC was provided by logos from respected and recognised organisations and the use of consistent colours, typography and images across the packaging and job aid reinforced that the medicine was genuine. Distinctive packaging has been used in public health distributions of artemisinin-based combination therapy to differentiate free medications supplied through the programme from those available through the private sector [49]. All categories of respondent in the current study were clear that the blister pack, dispensing pack and job aid materials must have a product identity that incorporates symbolism associated with malaria, such as a mosquito or an infant under an ITN. Use of such imagery presents an educational opportunity to reiterate the cause of malaria and the importance of prevention. As PMC was a novel concept for respondents in this study, such reinforcing messages will support understanding of the purpose of the intervention.

A previous study in Uganda indicated that specialised packaging for artemisinin-based combination therapy did not necessarily improve adherence, even when using pictorial representations to overcome limitations in literacy [50]. However, the incorporation of key messages relevant to the target group supported adherence [50]. This emphasizes the importance of field testing packaging in the specific context in which it will be used. In this study, the respondent feedback on the blister pack, dispensing box and job aid highlighted the context-specific expectations of users. They wanted to see equipment, people and situations that they were familiar with, and an accurate representation of their interpersonal relationships, e.g., the mother holding the child and administering the medication under supervision of the health worker. They identified areas where the images caused confusion, such as the use of a spoon for administration, or which had low acceptance, such as the colour black for the tablet. Additional areas for related educational opportunities were also highlighted, such as how to correctly hold a vomiting infant. There were also practical considerations, such as the preferred top opening dispensing box, perceived as secure and the larger format requested for the job aid. However, it is important that further work is conducted to evaluate the materials in the day-to-day practice of health providers during PMC implementation. This would indicate if there were gaps in the pre-implementation research, which could have been addressed, to inform the design of future similar studies into supportive drug packaging and educational resources.

Similar to the findings of the current study, recent reviews found that PMC was considered highly acceptable in malaria endemic communities [41, 51]. To accept PMC, communities must perceive that there is a need and believe that PMC will be a positive intervention to address this need. Where IPTp and SMC are implemented, there is also a need to differentiate PMC from these other preventive interventions. In Sierra Leone, the only country where PMC has been deployed, the intervention was generally accepted and perceived as efficacious, with the main challenges being logistical, such as access to water, crushing the tablets, and high staff turnover [21]. In the current study, PMC was perceived favourably, and the process of administration has been simplified using SP dispersible tablets, though access to clean water is required. Messages around improving acceptability included the representation of healthy infants who were smiling and well cared for. The role of the caregiver also needed to be emphasized as a key actor in the delivery of the medication. One crucial area was the explanation of adverse events. All respondents thought it was important to explain potential adverse events to caregivers, however, except for vomiting the pictorial representations were considered unhelpful. There was confusion with the symptoms of malaria and health professionals considered them to be alarming to caregivers. Rather, it was thought key to emphasize the verbal explanation of adverse events by health professionals to caregivers, initiating a more conversational approach that would enable concerns to be addressed and to encourage caregivers to attend the clinic should adverse events occur. The balance between provision of sufficient information on potential adverse events and raising unnecessary concerns has not been well examined, and this is an area which could benefit from further qualitative study, particularly in the context of strengthening pharmacovigilance systems [13, 40, 52, 53].

A key limitation of this study is that it did not consider mechanisms for pharmacovigilance. Also, there was no evaluation of actual implementation and the use of the materials in real-life practice. However, this will be included in the overall evaluation of the pilot studies and in a pilot project for PMC deployment in Togo. Although the range of participants was diverse, it was not comprehensive and specialised roles, such as pharmacovigilance, were not considered. It was clear that there were some key areas of divergence between the three countries, suggesting that use of these materials in other high-burden countries implementing PMC will require additional adaptations. Furthermore, suitable incentives for CHWs to deliver PMC and how their motivation would be secured and maintained were not evaluated.

## Conclusion

Appropriate drug packaging and job aids are required to support the uptake and acceptability of PMC as a new intervention. Simple modifications can have a profound effect on the perception of the intervention and influence acceptability. Through iterative quantitative investigation we developed PMC-specific materials suited to the local context and socio-cultural norms of the target population with the aim of increasing access to chemoprevention in children most at risk of malaria.

### Supplementary Information

Additional file 1.

## Data Availability

The datasets used and/or analysed during the current study are available from the corresponding author on reasonable request.

## Abbreviations

CHW: Community health worker
DOT: Directly observed therapy
FGD: Focus group discussion
IDI: In-depth individual interviews
IPTi: Intermittent preventive treatment in infants
IPTp: Intermittent preventive treatment in pregnancy
PMC: Perennial malaria chemoprevention
SMC: Seasonal malaria chemoprevention
SP: Sulfadoxine-pyrimethamine
WHO: World Health Organization
