# Supplementary material for: Field testing of user-friendly perennial malaria chemoprevention packaging in Benin, Côte d’Ivoire and Mozambique
Source: Malar J. 2024 May 21;23:157. doi: 10.1186/s12936-024-04977-0 (PMC11106929; doi:10.1186/s12936-024-04977-0)
Supplement: Supplementary file 1 — Additional file 1. [file 12936_2024_4977_MOESM1_ESM.pdf]

b. Interview guides

IDI Guide<br>HEALTH WORKERS,<br>COMMUNITY HEALTH<br>WORKERS	FIELD-TESTING OF USER-FRIENDLY INTERMITTENT PREVENTIVE TREATMENT FOR INFANT (IPTI) PACKAGING IN MALARIA-ENDEMIC COUNTRIES (BENIN- IVORY COAST- MOZAMBIQUE)

1. **Introduction and warm-up**
   - Introduce yourself and other members of the research team
   - Introduce of the research purpose and objectives.
   - Explain and complete informed consent.
   - Ask everyone to feel free to speak openly.
   - Explain how the tape recorder works and ask for permission to use it (anonymity).
   - Ask interviewees to introduce themselves
   -
2. **Importance of malaria at the community level**
   - Which diseases are of greatest concern to communities in this locality? (*Moderator ranks the top 3)*
   - If malaria is not mentioned, ask "Have you heard of malaria?
     - o What is the local name for malaria? (*Probe - for a name to distinguish fever from malaria*)
     - o What is the cause of malaria? (Record all responses)
   - In your community, which groups are most affected by malaria? (*Probe for infants, children*).
   - Which categories of children are most at risk for malaria
     - o Infants?
     - o Under 2 years old?
     - o Under 5 years of age?
     - o Under 10 years old?
   - What are the health risks associated with malaria for the category identified at most risk?
   - In your community, how are children protected from malaria?
     - o Moderator, please list all actions mentioned.
     - o For each action listed, probe for positive/negative experience with that action.
3. **Preventing malaria in children with IPT**
   - Do you know about how to protect children from malaria? *Ask for details*
     - o Is IPT listed as prevention mean
   - What types of IPT do you know about for children? Probe
     - o SMC (Children under five years of age),
     - o IPTi (infants)
   - How do you feel about children taking medicine to prevent malaria? (*Probe for positive and negative views*)
   - Have you ever been involved in prescribing or distributing these IPT drugs to children? Describe and explain
     - o benefit of IPT and SP in protecting infants from malaria?
     - o What are the facilitators/constraints in distributing drugs to children
     - o What are the main challenges? How can they be overcome?
   - What do you think about the medicines prescribed to children to prevent malaria?
     - o In your experience, is this medication effective in preventing malaria?
     - o How do parents feel about this medication and giving it to children?
   - IPTi is currently reserved for infants (less than 11 months). In your opinion, is it relevant and feasible to extend the distribution scheme to 2 years of age? Why or why not? Explain
     - o feasibility of implementation: factors and barriers
     - o Community adherence : political and socio-cultural specificities
   - As you may know, today SP is given to children at the time of vaccination (EPI). In the future, community health workers could distribute it at the community level, ensuring that children consume the tablets in front of them.
     - o In your opinion, is this involvement feasible? Probe for details
     - o Do you see any benefits to involving CHWs in IPT among infants? Probe for details
     - o What might be the constraints of involving CHWs in prescribing IPT?
     - o Do you think women would find it acceptable that community workers to administer IPT to their children? Probe for more details

   - As you know, some children are given SP to protect them from malaria and others do not.

- o What do you think are the reasons for use? (free list). *Probe for reasons, including those related to SP.*
- o In your opinion, what are the reasons for non-use? (Free list). *Probe for reasons, including those related to SP.*

Moderator should thank participants for their valuable comments.

4. Evaluation / Testing of SP packaging

- I will now show you a sample package of SP with a new pediatric formulation. As you can see, it contains tablets that will be given to children to take in front of the health worker.
-
- First, I am going to show you the package that contains dispersible SP tablets and ask you to take the time to look at them carefully. I ask you to examine each one carefully so that we can discuss about it. We want to explore with you how we can make them better, more attractive, suitable, and acceptable to facilitate the administration of SP-IPTi.

GIVE THE RESPONDENT A FEW MINUTES TO OBSERVE

- After observing the SP packaging, what are your first impressions and comments?

ENCOURAGE THE RESPONDENT TO GIVE HIS/HER VIEWS WITHOUT INFLUENCING HIM/HER

- Can you tell me what you understand at first glance about this package? (information conveyed, related to the drug)
- From your point of view, what are the "pros" / "cons" of such a package?

***5.* General appreciation of the packaging and the SP product**

- What is the overall appeal of the SP packaging format?
  - o Aesthetics (shape, color, presentation) paper quality etc.)
  - o User-friendliness
- Are the shape, color, images represented acceptable to you? For what reasons?
- What are the advantages/disadvantages of the SP packaging? Probe:
  - o Quality? Safety? Efficacy?
  - o Drug identity?
  - o Visual?
- Is this package suitable for prescribing medicines to children?
- What attributes do you think the package should have to facilitate your community outreach work? *(Probe for desired graphic options, images, format, directions)*
- Other important elements to include in the package?
- What would be needed to be improved in the package to make it more user-friendly and facilitate your work as a community liaison?
-

6. Appreciation of the contents of packaging prototype

- What do you think of the way the SP medication is presented in this blister pack? Probe the comments
- Do you think the presentation of SP in this type of blister pack is a significant difference? Probe for reasons
- Can the blister pack help give the drug a specific identity? If yes, in what way? If not, why not?
- Looking at the information conveyed by the packaging,
  - o Do you think it is a good visual aid or tool? In what way? Explain
  - o Do you think it is a good reminder of proper medication preparation and administration?
- Do you think that providing SP in this package would motivate parents to accept SP for children? If yes, in what way? If not, why not?

V. Understanding, appreciation of information messages conveyed by IEC tools (leaflets, job aids, etc.)

- Are the key messages on IEC tools clearly understood by health care providers (at the facility or community level) and caregivers?
- When you look at this brochure, what is the main message that strikes you and that you remember? *Probe for details* :
  - o What strikes you most about these images? Probe for details.
  - o What did you like best? Probe for reasons.
  - o What did you like least / not at all? Probe for reasons
- Do you think the illustration is appropriate?
  - o If yes, why? If no, why not?
  - o Are there any textual or visual elements that pose a problem for understanding?
  - o Are there any textual or visual elements that should aid comprehension (if so, which ones)?
  - o Can you suggest any changes to the illustration?
  - o
- What did you understand about each image and the key messages of the insert? *Systematically review each illustration and probe for understanding of :*
  - o The dosing information,
  - o Information on preparing the medication;
  - o Directions for administering the IPTi-SP preparation,
  - o Danger signs;
  - o Dosage regimen, when to take the next dose

- o When/where to consult in case of side effects
- Do you think the IEC tools are an acceptable and appropriate educational tool for health workers and caregivers?
  - o Do you think the information tools presented would help health care workers understand IPTi and encourage parents to accept it for their children?
  - o Do you think you would better to learn and remember the messages, instructions, or procedures about IPTi with these tools?
  - o
- What do you think of the messages conveyed in the IEC tools?
  - o Are they clear for a community outreach worker?
  - o Do they meet the expectations and information needs that you consider useful for your work in prescribing IPTi?
  - o Are there additional information needs that would benefit from being introduced into the prototype packaging and IEC tools?
- Which of the different versions of job-aid and inserts that I have shown you do you prefer?
  - o Look carefully and explain why?
  - o What changes do you think should be made to this preferred version

**IDI Guide<br>HEALTH AUTHORITIES**	**FIELD-TESTING OF USER-FRIENDLY INTERMITTENT PREVENTIVE TREATMENT FOR INFANT (IPTI) PACKAGING IN MALARIA-ENDEMIC COUNTRIES (BENIN- IVORY COAST- MOZAMBIQUE)**

1. **Introduction and warm-up**
   - Introduce yourself and other members of the research team
   - Introduce of the research purpose and objectives.
   - Explain and complete informed consent.
   - Ask everyone to feel free to speak openly.
   - Explain how the tape recorder works and ask for permission to use it (anonymity).
   - Ask interviewees to introduce themselves
2. **Malaria prevention among children status in the area of responsibility**
   -
   - Health problems more experienced by children in the area of responsibility
   - Current infant malaria prevention strategies underway in the district
     - o Chemo prevention of seasonal malaria during the rainy season
     - o Intermittent preventive treatment for infants (IPTi)
   -
   - What are the experiences in the implementation of the CPS: challenges, opportunities
     - o Challenges and opportunities related to the current malaria prevention policy (SMC)
     - o SP drugs
     - o Facilitators/constraints in distributing medicines to children
   -
   - As a key implementing partner concerned with the issue of malaria control, could you share your perspective on the IPT intervention:
     - o Benefits of IPT and SP in protecting infants from malaria
     - o Challenges in adopting this strategy at the national level
     - o Views on the SP drug prescribed to infants to prevent malaria?
     - o In your experience, is this drug effective in preventing malaria?
     - o How do parents feel about this drug and giving it to children?
     - o
   - TPIn is currently reserved for infants. In your opinion, is it appropriate and feasible to extend the distribution schedule to 2 years of age? Why or why not? Explain
     - o Feasibility of implementation: drivers and barriers
     - o Community buy-in: political and socio-cultural specifics
     - o
   - As you may know, today SP is given to children at the time of immunization (EPI). In the future, community health workers could distribute it at the community level, ensuring that children consume the tablets in front of them.
     - o In your opinion, is this involvement feasible? Probe for details
     - o Do you see any benefits to involving CHWs in IPT for infants? Probe

- What might be the constraints of their involvement in prescribing IPT?
- Do you think women would find it acceptable for community workers to administer IPT to their children? Probe for more details
- o

3. **Evaluation / Testing of SP packaging**

- I will now show you a sample package of SP with a new pediatric formulation. As you can see, it contains tablets that will be given to children to take in front of the health worker. I ask you to examine each one carefully so that we can discuss about it. We want to explore with you how we can make them better, more attractive, suitable, and acceptable to facilitate the administration of SP-IPTi.

- **General appreciation of the packaging and the SP product**
  - What is the overall appeal of the SP packaging format?
    - Aesthetics (shape, color, presentation) paper quality etc.)
    - User-friendliness
  - Are the shape, color, images represented acceptable to you? For what reasons?
  - What are the advantages/disadvantages of the SP packaging? Probe:
    - Quality? Safety? Efficacy?
    - Drug identity?
    - Visual?
  - Is this package suitable for prescribing medicines to children?
  - What attributes do you think the package should have to facilitate your community outreach work? *(Probe for desired graphic options, images, format, directions)*
  - Other important elements to include in the package?
  - What would be needed to be improved in the package to make it more user-friendly and facilitate your work as a community liaison?
  - To your knowledge, are there any national standards/recommendations/regulations to be considered in packaging artwork development?
  - o
- ***Appreciation of the contents of packaging prototype***
  - What do you think of the way the SP medication is presented in this blister pack? Probe the comments
  - Do you think the presentation of SP in this type of blister pack is a significant difference? Probe for reasons
  - Can the blister pack help give the drug a specific identity? If yes, in what way? If not, why not?
  - Looking at the information conveyed by the packaging,
    - Do you think it is a good visual aid or tool? In what way? Explain
    - Do you think it is a good reminder of proper medication preparation and administration?
  - Do you think that providing SP in this package would motivate parents to accept SP for children? If yes, in what way? If not, why not?
- **Understanding, appreciation of information messages conveyed by IEC tools (leaflets, job aids, etc.)**
  - When you look at this package/brochure, what is the main message that strikes you and that you remember? Probe for details
  - Do you think the images and messages presented can be understood by health workers?
  - Do you think the messages presented here are the most important and cover the information health workers need to prescribe and promote IPTi?
    - ✔ Are they clear for a community outreach?
    - ✔ Do they meet the expectations and information needs you feel are useful for the work of prescribing IPTi?
    - ✔ Are there additional information needs that would benefit from being introduced into the prototype packaging and IEC tools?
  - Can the messages presented visually in this package help to better prescribe and explain instructions for SP preparation and prescription?
  - Do you think the illustrations are appropriate?
    - If yes, why? If no, why not?
    - Textual or visual elements that are problematic to understand (if yes, which ones)?
    - Are there any textual or visual elements that aid comprehension (if so, which ones)?
    - Can you suggest any changes to the illustration
  - Do you think the IEC package and tools are an acceptable and appropriate educational tool for health workers and caregivers?

4. **Recommendations for packaging and IEC tools improvement to better promote the prescription of IPTi at the community level**

5. **Assessment of the community workers role in the distribution and promotion of the IPTi packaging**

There are no new distribution mechanisms in place yet, but I want to discuss with you about possible alternatives scenarios (e.g. with the support of community relays. According to this scheme, community health workers are responsible for carrying out the distribution and ensuring the consumption of doses of SP in directly observed treatment

- Is their involvement acceptable and feasible?
- Do you think these CHW are legitimate and have capacities to distribute and supervise SP tablets taking by children? Please explain
- What are the benefits of involving these actors, the opportunities for their involvement, but also the potential risks and constraints?
- If community health workers were to be involved in the distribution and supervision of the use of the packaging, what should be improved or taken as an accompanying measure?

Guide for Individual Interview CAREGIVERS	FIELD-TESTING OF USER-FRIENDLY INTERMITTENT PREVENTIVE TREATMENT FOR INFANT (IPTI) PACKAGING IN MALARIA-ENDEMIC COUNTRIES (BENIN- IVORY COAST- MOZAMBIQUE)

1. **Introduction and warm-up**
   - Interviewer introduces himself and his collaborator.
   - Introduction of the field testing and its purpose and objectives.
   - Explains informed consent and fill out informed consent form
   - Requests the respondent to speak openly. Their honest opinion is important even if it may be different from what others think
   - Explain the tape recorder and request permission to use it (explain that anonymity is guaranteed)
   - Ask respondents to introduce themselves. (Probe for name, age, occupation, marital status)
2. **Importance of malaria at household / community**
   - What diseases are your most worried about? **Moderator rank top 3**
     - o If malaria is not mentioned, ask "Have you heard of malaria"?
     - o What is the local name for malaria? (**Probe – for a distinguishing name between fever & malaria**)
   - In your family who is most affected by malaria? **Moderator please list all mentioned.**
   - What are the health risks related to malaria? (probe For children)
   - In your family, how do people to protect children from malaria? **Moderator please list all mentioned.**
     - o Probe for positive / negative experience with it.
3. **Preventing malaria in children with IPT**
   - Do you know about how IPT as mean of preventing children from malaria? *Ask for details*
   - What types of IPT do you know about for children? Probe
     - o SMC (Children under five years of age),
     - o IPTi (infants)
   - How do you feel about children taking medicine to prevent malaria? (*Probe for positive and negative views*)
     - o benefit of IPT and SP in protecting infants from malaria?
     - o What are the facilitators/constraints in distributing drugs to children
   - What do you think about the medicines prescribed to children to prevent malaria?
     - o In your experience, is this medication effective in preventing malaria?
     - o How do you feel about this medication and giving it to your children?
   - As you know, some children are given SP to protect them from malaria and others do not.
     - o What do you think are the reasons for use? (free list). *Probe for reasons, including those related to SP.*
     - o In your opinion, what are the reasons for non-use? (Free list). *Probe for reasons, including those related to SP.*
4. **Assessment / Testing of the SP packaging**

   I will now show you a sample package of SP with a new pediatric formulation. As you can see, it contains tablets that will be given to children to take in front of the health worker. First, I am going to show you the package that contains dispersible SP tablets and ask you to take the time to look at them carefully. I ask you to examine each one carefully so that we can discuss about it. We want to explore with you how we can make them better, more attractive, suitable, and acceptable to facilitate the administration of SP-IPTi.

   GIVE THE RESPONDENT A FEW MINUTES TO OBSERVE

   After observing the SP packaging, what are your first impressions and comments?

ENCOURAGE THE RESPONDENT TO GIVE HIS/HER VIEWS WITHOUT INFLUENCING HIM/HER

- Can you tell me what you understand at first glance about this package? (information conveyed, related to the drug)
- From your point of view, what are the "pros" / "cons" of such a package?

- **General appreciation of the packaging and the SP product**
  - *o* What is the overall appeal of the SP packaging format?
    - ▪ Aesthetics (shape, color, presentation) paper quality etc.)
    - ▪ User-friendliness
  - o Are the shape, color, images represented acceptable to you? For what reasons?
  - o What are the advantages/disadvantages of the SP packaging? Probe:
    - ▪ Quality? Safety? Efficacy?
    - ▪ Drug identity?
    - ▪ Visual?
  - o What would be needed to be improved in the package to make it more user-friendly?

- o
- **Appreciation of the contents of packaging prototype**
    - o What do you think of the way the SP medication is presented in this blister pack? Probe the comments
    - o Do you think the presentation of SP in this type of blister pack is a significant difference? Probe for reasons
    - o Can the blister pack help give the drug a specific identity? If yes, in what way? If not, why not?
    - o Do you think that providing SP in this package would motivate parents to accept SP for children? If yes, in what way? If not, why not?
- **Understanding, appreciation of information messages conveyed by IEC tools (leaflets, job aids, etc.)**
    - When you look at this brochure, what is the main message that strikes you and that you remember? *Probe for details* :
        - o What strikes you most about these images? Probe for details.
        - o What did you like best? Probe for reasons.
        - o What did you like least / not at all? Probe for reasons
    - Do you think the illustration is appropriate?
        - o If yes, why? If no, why not?
        - o Are there any textual or visual elements that pose a problem for understanding?
        - o Are there any textual or visual elements that should aid comprehension (if so, which ones)?
        - o Can you suggest any changes to the illustration?
        - o
    - What did you understand about each image and the key messages of the insert? *Systematically review each illustration and probe for understanding of :*
        - o The dosing information,
        - o Information on preparing the medication;
        - o Directions for administering the IPTi-SP preparation,
        - o Danger signs;
        - o Dosage regimen, when to take the next dose
        - o When/where to consult in case of side effects

5. **Assessment of the community workers role in the distribution and promotion of the IPTi packaging**

There are no new distribution mechanisms in place yet, but I want to discuss with you about possible alternatives scenarios (e.g. with the support of community relays. According to this scheme, community health workers are responsible for carrying out the distribution and ensuring the consumption of doses of SP in directly supervised treatment

- o Is their involvement acceptable and feasible?
- o Do you think these CHW are legitimate and have capacities to distribute and supervise SP tablets taking by children? Please explain
- o How would feel about collecting SP from the community health worker? *Probe for details*
- o Would you be willing to take SP under the supervision of community health workers?
    - ▪ If yes, why and what reasons (related to CHW) can motivate you
    - ▪ If no, why not and what reasons (related to CHW) can be constraints for you?

The interviewer should thank the respondent for their valuable comments.
